# High density and proximity of CD8 ^+^ T cells to tumor cells are correlated with better response to nivolumab treatment in metastatic pleural mesothelioma

**DOI:** 10.1111/1759-7714.14981

**Published:** 2023-05-30

**Authors:** Yuting Yin, Rie Sakakibara, Takayuki Honda, Susumu Kirimura, Pissacha Daroonpan, Masashi Kobayashi, Kohei Ando, Hideki Ujiie, Tatsuya Kato, Kichizo Kaga, Takahiro Mitsumura, Ryoji Nakano, Hiroyuki Sakashita, Shinichi Matsuge, Hironori Ishibashi, Takumi Akashi, Yasuhiro Hida, Takao Morohoshi, Miyuki Azuma, Kenichi Okubo, Yasunari Miyazaki

**Affiliations:** ^1^ Department of Respiratory Medicine Tokyo Medical and Dental University Tokyo Japan; ^2^ Department of Pathology Tokyo Medical and Dental University Tokyo Japan; ^3^ Department of Molecular Immunology Tokyo Medical and Dental University Tokyo Japan; ^4^ Department of Thoracic Surgery Tokyo Medical and Dental University Tokyo Japan; ^5^ Department of Thoracic Surgery Yokosuka Kyosai Hospital Yokosuka Japan; ^6^ Department of Thoracic Surgery Hokkaido University Hospital Sapporo Japan; ^7^ Department of Pulmonary Immunotherapeutics Tokyo Medical and Dental University Tokyo Japan; ^8^ Department of Respiratory Medicine Hokkaido Kin‐Ikyo Chuo Hospital Sapporo Japan; ^9^ Department of Chemotherapy Yokosuka Kyosai Hospital Yokosuka Japan; ^10^ Department of Thoracic Surgery Hokkaido Kin‐Ikyo Chuo Hospital Sapporo Japan

**Keywords:** immune checkpoint inhibitor (ICI), multiplex immunofluorescence (mIF), nivolumab, pleural mesothelioma, tumor microenvironment (TME)

## Abstract

**Background:**

The efficacy of immune checkpoint inhibitors (ICIs) in pleural mesothelioma has recently been established. The response to ICIs can be predicted by quantitative analysis of cells and their spatial distribution in the tumor microenvironment (TME). However, the detailed composition of the TME in pleural mesothelioma has not been reported. We evaluated the association between the TME and response to ICIs in this cancer.

**Methods:**

A retrospective analysis of 22 pleural mesothelioma patients treated with nivolumab in different centers was performed using surgical specimens. Four patients had a partial response to nivolumab (response group) and 18 patients had stable or progressive disease (nonresponse group). The number of CD4, CD8, FoxP3, CK, and PD‐L1 positive cells, cell density, and cell‐to‐cell distance were analyzed by multiplex immunofluorescence.

**Results:**

PD‐L1 expression did not differ significantly between the response and nonresponse groups. The density of total T cells and of CD8^+^ T cells was significantly higher in the response than in the nonresponse group. CD8^+^ T cells were more clustered and located closer to tumor cells, whereas regulatory T cells were located further from tumor cells in the response than in the nonresponse group.

**Conclusions:**

High density and spatial proximity of CD8^+^ T cells to tumor cells were associated with better response to nivolumab, whereas the proximity of regulatory T cells to tumor cells was associated with worse response, suggesting that the distinct landscape of the TME could be a potential predictor of ICI efficacy in pleural mesothelioma.

## Graphical abstract

The distribution of the tumor microenvironment (TME) in pleural mesothelioma has not been reported. We evaluated the association between the TME and response to nivolumab in pleural mesothelioma. High density and spatial proximity of CD8^+^ T cells to tumor cells were associated with better response to nivolumab. Our research suggests that the distinct landscape of the TME could be a potential predictor of ICI efficacy in pleural mesothelioma.

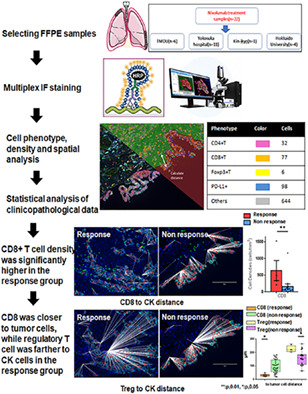

## INTRODUCTION

Pleural mesothelioma is a rare and aggressive tumor with an incidence of a few cases per million people,^1^ and has a poor prognosis, with a 5‐year survival rate of <10%.^2^ Recent clinical studies have demonstrated the efficacy of immune checkpoint inhibitors (ICIs) in delaying tumor progression in patients with advanced or recurrent pleural mesothelioma.^3^, ^4^ Programmed death ligand 1 (PD‐L1) expression was controversial in predicting ICI efficacy when detected by immunohistochemical analysis of surgical specimens.^5^, ^6^

The type, density, and location of immune cells in the tumor microenvironment (TME) affects the efficacy of ICIs against solid tumors.^7^, ^8^ The baseline T cell density and location at the infiltrative margin predict the outcome of metastatic melanoma patients receiving PD‐1‐targeted therapy.^9^

The density of tumor‐infiltrating lymphocytes (TILs) as well as their spatial distribution in the TME can be analyzed by multiplex immunofluorescence (mIF).^10^, ^11^, ^12^, ^13^ In tongue squamous cell carcinoma, CD8^+^ T cell or CD4^+^Foxp3^+^ regulatory T cell (Treg) infiltration was identified using mIF.^14^ The proximity of Tregs to tumor cells is significantly negatively associated with survival in non‐small cell lung cancer (NSCLC).^15^

In immunotherapy for pleural mesothelioma, there is no evidence of an association between the TME and the response to ICIs or immune‐related adverse events (irAEs). In this study, we investigated the clinical impact of the TME in pleural mesothelioma patients treated with ICIs. We retrospectively evaluated the efficacy of nivolumab in patients with pleural mesothelioma and performed an integrated analysis of the TME, including PD‐L1 expression and the density and spatial distribution of TILs, using mIF to determine the association between the TME and the efficacy of ICIs.

## METHODS

### Patient selection and data collection

Twenty‐two patients with pleural mesothelioma who had relapsed after surgery and were treated with nivolumab between August 2016 and October 2021 were enrolled from four hospitals in Japan: Tokyo Medical and Dental University (TMDU) Hospital, Kin‐ikyo Chuo Hospital, Hokkaido University Hospital, and Yokosuka Kyosai Hospital. All the samples were surgical samples as first‐line treatment, not samples taken immediately prior to nivolumab. This study was approved by the Ethics Committee of each participating institution (M2020‐224‐01). In addition, patients with autoimmune disease or interstitial pneumonia were excluded as nivolumab selection criteria. In this study, all patients met the selection criteria. As this was an observational study, informed consent was not obtained from individual patients. Instead, the information was disclosed on the websites of the Bioethics Research Center and the Department of Respiratory Medicine at TMDU.

Clinical data were collected from the medical records of each patient and included age, sex, smoking history, staging, histology, treatment‐related information concerning nivolumab, such as response to nivolumab, and irAEs. The TNM stage of pleural mesothelioma was defined according to the International Mesothelioma Interest Group classification^16^ and histological types were defined according to the World Health Organization classification.^17^ Response to nivolumab was evaluated by pulmonologists and/or thoracic surgeons at each hospital according to the methodology described in the modified Response Evaluation Criteria in Solid Tumors (mRECIST).^18^ The best response was evaluated and categorized as a complete response (CR), partial response (PR), stable disease (SD), or progressive disease (PD). The patients were divided according to nivolumab response into a “response” group, which included patients showing CR or PR, and a “nonresponse” group, which included those showing SD or PD. Progression‐free survival (PFS) was defined as the beginning of initial nivolumab administration until the SD response was observed. The definition and grading classification of irAEs were based on the Common Terminology Criteria for Adverse Events (CTCAE) version 5.0.

### Tissue selection

For each tumor, all available hematoxylin and eosin (H&E) slides were reviewed by a pathologist, and the single most representative lesion was selected. Two pathologists (SK, RS) independently reviewed the histology for each patient and divided tissues into intra‐ and extratumoral areas. If the opinions of two pathologists did not agree, the one that agreed with the opinion of the third pathologist (YY) was adopted. The intratumoral areas, including tumor nests containing tumor cells and intratumor stromal components, were defined as the “central tumor” (CT),^19^, ^20^, ^21^ and the extratumoral areas were defined as the “invasive margin” (IM)^22^ (Figure S1). Cell counts and intercellular distances were analyzed in five areas: three CT areas and two IM areas. The final values of density and distance for each individual were defined as the average value of the total CT or IM areas.

### Opal multiplex immunofluorescence (mIF)

The mIF analysis was performed using 4‐μm‐thick formalin‐fixed, paraffin‐embedded (FFPE) sections (Figure S2a). mIF was performed using an Opal seven‐color immunohistochemistry (IHC) kit (Akoya Biosciences). FFPE sections were fixed in 10% neutral‐buffered formalin (NBF) before paraffin block, then deparaffinized, rehydrated, and fixed one more time in 10% NBF buffer for 20 min before peroxide blocking. Antigen retrieval was performed using either citrate buffer or 0.1% sodium azide (pH 9) buffer (Nichirei Biosciences) and microwave treatment for 15 min. The slides were washed with TBST/0.5% Tween (three times, 2 min each) and incubated with 3% H_2_O_2_ for 10 min. Then, slides were incubated with the following primary antibodies: CD4 (4B12, Nichirei Biosciences, Ready to use/Opal 520), CD8 (C8/144B, Nichirei Biosciences, Ready to use/Opal 570), PD‐L1 (E1J2J, Cell Signaling Technology; 1:200/Opal 540), FoxP3 (236A/E7, Abcam; 1:100/Opal 620), pan‐CK (C11, Cell Signaling Technology; 1:500/Opal 690). CD8/CD4/PD‐L1/pan‐CK was used for membrane staining, FoxP3 for nuclear staining. Tyramide signal amplification solution was applied after the corresponding secondary HRP‐conjugated polymer for each mAb from the Opal seven‐color IHC kit. Nuclei were stained with spectral 4′, 6‐diamino‐2‐phenylindole (DAPI) and mounted using ProLong Gold Antifade Reagent (Cell Signaling Technology).

### Image analysis

Five representative pictures for each patient were captured at 200× magnification using the Mantra platform (Perkin Elmer; version 1.0.3). As described previously, inForm Image Analysis software (PerkinElmer, version 2.4.11)^14^, ^23^ was used to recognize and characterize different cell phenotypes in tumor tissues (Figure S2b). Each cell was classified using the following surface markers: CD8^+^ for cytotoxic T cells (CD8^+^); CD4^+^FoxP3^−^ for conventional T cells (Tcons); CD4^+^FoxP3^+^ for regulatory T cells (Tregs); pan‐CK^+^ for tumor cells (CK); PD‐L1^+^pan‐CK^+^ for PD‐L1^+^ tumor cells (PD‐L1^+^CK). With the exception of CD8^+^ T cells and pan‐CK^+^ tumor cells, which require a single cell marker for identification, cells were identified by a combination of two cell markers as follows: PD‐L1^+^CK (PD‐L1^+^pan‐CK^+^) (Figure S2c), Tcon (CD4^+^Foxp3^−^), and Treg (CD4^+^Foxp3^+^) (Figure S2d). Cell density was calculated using the following formula: number of cells in the area divided by the whole tissue area (cells/mm^2^). Phenoptr and phenoptrReports (R, version 4.1.1) were used for identification of cellular subsets and spatial analysis including cell–cell distance. Distance between two cell subtypes was calculated using the x‐ and y‐coordinates from the inForm raw data and further calculated by finding per‐cell nearest neighbor distance using phenoptr. The distance between each cell and its nearest cell of both the same cell phenotype and the other cell phenotype were automatically calculated and the mean was determined.

### Statistical analysis

Statistical analyses of patient characteristics were performed using the Mann–Whitney U test and Fisher's exact test for comparisons of continuous variables, where appropriate. Kaplan–Meier curves and log‐rank test were used for analysis of PFS using SPSS 19 software (IBM SPSS Statistics for Windows). Uni‐ and multivariate analysis was performed using the Cox proportional hazard model to calculate hazard ratio (HR) and 95% confidence interval (CI) for each potential risk factor. A *p*‐value <0.05 was considered statistically significant.

## RESULTS

### Patient characteristics

Patient characteristics are detailed in Table 1. Four patients were included in the response group and 18 patients in the nonresponse group. The median age of the entire cohort was 65 years, and the response group was significantly younger than the nonresponse group (*p* = 0.005). In the cohort, the pathological features of the epithelial type were dominant (*n* = 21, 96%), because generally only epithelial mesotheliomas are resected. However, biphasic mesotheliomas may also be resected if the initial biopsy does not identify a sarcomatoid phase or because the patient's performance status is high and young. A total of 82% of patients had a smoking history and 73% had asbestos exposure, similar to previous studies.^24^ There were no significant differences in surgical procedure, histology, history of smoking, or asbestos exposure between the two groups.

**TABLE 1:** Patient characteristics.

Patient characteristics	Total (*n* = 22)	Response group (CR and PR, *n* = 4)	Non‐response group (SD and PD, *n* = 18)	*p*‐value
Age, median (range)	65 (43–80)	50 (43–63)	68 (51–80)	0.005
Sex				1.000
Male	21 (96%)	4	17	
Female	1 (4%)	0	1	
Histology				0.182
Epithelioid	21 (96%)	3	18	
Biphasic	1 (4%)	1	0	
Smoking history				
Current	18 (82%)	3	15	1.000
Never	4 (18%)	1	3	
Asbestos exposure				1.000
Current	16 (73%)	3	13	
Never	4 (18%)	1	3	
NE	2 (9%)	0	2	
Stage				
III, IV	8 (36%)	3	5	0.117
I, II	14 (64%)	1	13	
Preoperative therapy				0.535
Chemotherapy	5 (23%)	0	5	
None	17 (77%)	4	13	

*Note*: Data are presented as numbers (%) unless otherwise specified. The *p*‐value was calculated using the Mann–Whitney U test or Fisher's exact test, where appropriate.

Abbreviations: CR, complete response; NE, not evaluated; PD, progressive disease; PR, partial response; SD, stable disease.

### Quantitative analysis of the TME and correlation with nivolumab efficacy in pleural mesothelioma

**FIGURE 1:**
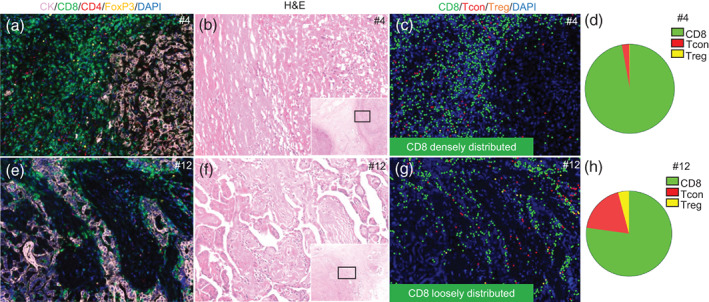
Multiplex immunofluorescence (mIF), hematoxylin and eosin (H&E) staining, and cell phenotype analyses in two representative cases (#4 and #12). (a, e) Five‐color mIF showing three T‐cell subsets and CK staining. Images are shown at 200× magnification. Fluorescence: CK (pink)/CD8 (green)/CD4 (red)/Foxp3 (yellow)/DAPI (blue). (b, f) H&E staining at 40× magnification and 200× magnification. (c, g) Cell phenotype dot plots of CD8/Tcon/Treg based on the inForm software learning algorithm. Images are shown at 200× magnification. Dots: CD8^+^ T cells (green), CD4^+^Foxp3^−^ conventional T cells (Tcon; red), CD4^+^Foxp3^+^ regulatory T cells (Treg; yellow). (d, h) Pie chart showing the proportions of three T‐cell subsets in each patient: CD8^+^ T, green; Tcon, red; and Treg, yellow. Circle size indicates total T cell density.

**FIGURE 2:**
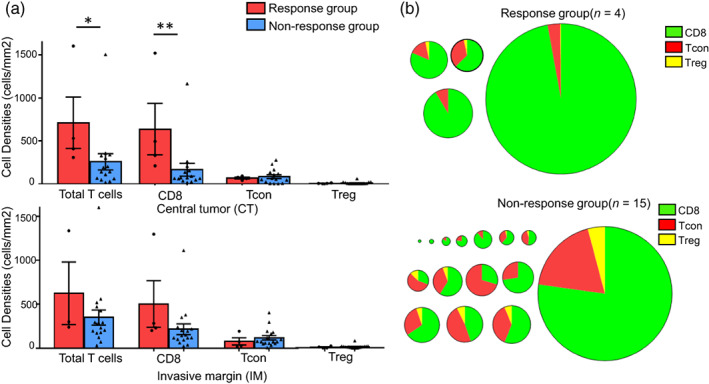
Analysis of T cell density and proportion in the response and nonresponse groups. (a) Box plot of the densities of total TILs (CD8^+^+CD4^+^), CD8, Tcons, and Tregs. Densities were evaluated as the number of positive cells per mm^2^. *p*‐value was calculated using the Mann–Whitney U test. *p < 0.05, **p < 0.01. Tcons: CD4^+^Foxp3^−^ conventional T cells. Tregs: CD4^+^Foxp3^+^ regulatory T cells. (b) Proportions of CD8, Tcons, and Tregs are presented as a pie chart in the central tumor (CT) in the response and nonresponse groups. CD8 in green, Tcons in red, and Tregs in yellow. Circle size indicates total T cell density.

To better characterize the three T cell subpopulations, quantitative analysis was performed. Two representative cases of different T cell distributions are shown in Figure 1, and CD8 was densely (Figure 1c) or loosely (Figure 1g) distributed. The individual T cell details are shown in Figure 2. Generally, total T cell infiltration was comparable between the CT and the IM (349.2 cells/mm^2^ in the CT vs. 359.6 cells/mm^2^ in the IM, Figure 2a). The densities of total T cells and CD8^+^ T cells were significantly higher in the response than in the nonresponse group (Table 2, 711.3 cells/mm^2^ vs. 252.7 cells/mm^2^, *p* < 0.05; 637.8 cells/mm^2^ vs. 161.4 cells/mm^2^, *p* < 0.01, respectively), whereas no significant differences in Tcons or Tregs were observed in the CT (Figure 2a). There were no significant differences in the IM regardless of the T cell subpopulation considered (Figure 2a). Data were plotted as a pie chart to determine the proportion of T cell subsets in each individual. The results showed that CD8^+^ T cells predominated among all T cells (68%), whereas Tcons accounted for 29% and Tregs for 3%. The mean proportion of CD8 was 83% (range, 60%–97%) in the response group and 64% (range, 31%–100%) in the nonresponse group. Likewise, the mean proportions of Tcons and Tregs were 15% (range, 3%–36%) and 2% (range, 0%–3%) in the response group, and 32% (range, 0%–61%) and 4% (range, 0%–11%) in the nonresponse group, respectively. There were no differences in the proportions of the three T cell subpopulations between the response and nonresponse groups (Figure 2b). No significance was observed in the IM (data not shown).

**TABLE 2:** T cell densities between the response and the nonresponse groups.

Categories	Mean (range) (cells/mm^2^)	
	Response	Non‐response
Total T (central tumor)	711.3 (305.9–1602.3)	252.7 (21.5–1504.1)
Total T (invasive margin)	581.1 (241.1–1335.7)	307.4 (12.6–1599.4)
CD8 (central tumor)	637.8 (209.1–1520.9)	161.4 (21.5–1166.4)
CD8 (invasive margin)	503.7 (202.0–1297.8)	196.3 (12.6–1112.6)
Tcon (central tumor)	67.5 (40.2–91.2)	81.3 (0.0–276.9)
Tcon (invasive margin)	78.6 (16.8–193.6)	99.0 (0.0–405.5)
Treg (central tumor)	6.1 (0.0–13.0)	10.0 (0.0–60.8)
Treg (invasive margin)	8.4 (0.0–22.4)	12.1 (0.0–81.4)

Abbreviations: Tcon, conventional T cells; Treg, regulatory T cells.

### Lack of correlation between PD‐L1 expression and ICI efficacy

**FIGURE 3:**
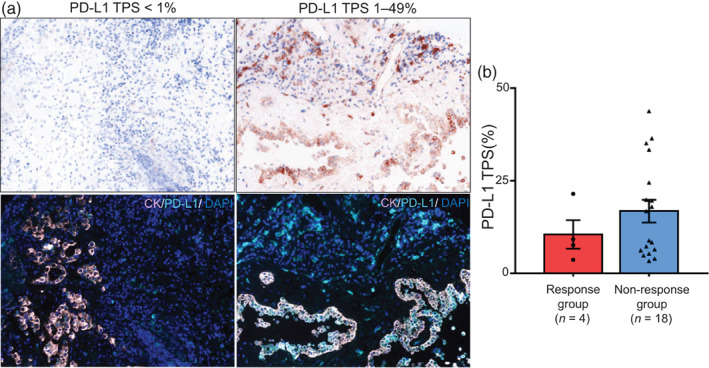
Images and analysis of PD‐L1 expression in the response and nonresponse groups. (a) Representative hematoxylin and eosin (H&E) staining and tumor proportion score (TPS) with PD‐L1 of <1% (left) and 1%–49% (right). Images are shown at 200× magnification. Fluorescence: CK (pink)/PD‐L1 (cyan)/DAPI (blue). (b) Distribution of PD‐L1 TPS in the response group (*n* = 4) and nonresponse group (*n* = 18). The *p*‐value was calculated using the Mann–Whitney U test.

To obtain basic information about immune checkpoint molecules in pleural mesothelioma, PD‐L1 positivity was assessed using mIF. We used a specific anti‐PD‐L1 antibody, E1J2J, which was selected because of its availability and was confirmed to show high concordance with the clinically‐used anti‐PD‐L1 antibody 22C3 (Figure S3), as reported in a previous study.^25^ Tumor proportion score (TPS), which calculates the percentage of tumor cells expressing PD‐L1, was used to assess PD‐L1 expression (Figure 3a). Consistent with previous research in pleural mesothelioma^24^ and NSCLC,^26^, ^27^, ^28^ a TPS of ≥1% was considered to indicate PD‐L1 positivity. Positive PD‐L1 expression was detected in all 22 samples in this study. The mean TPS was 11% in the response group and 17% in the nonresponse group. TPS ≥50% did not exist with the maximum TPS of 44% in this study. There was no significant difference in PD‐L1 expression between the response and nonresponse groups (Figure 3b).

### Spatial distribution of CD8 ^+^ T cells and Tregs to tumor cells predicts the response to nivolumab

**FIGURE 4:**
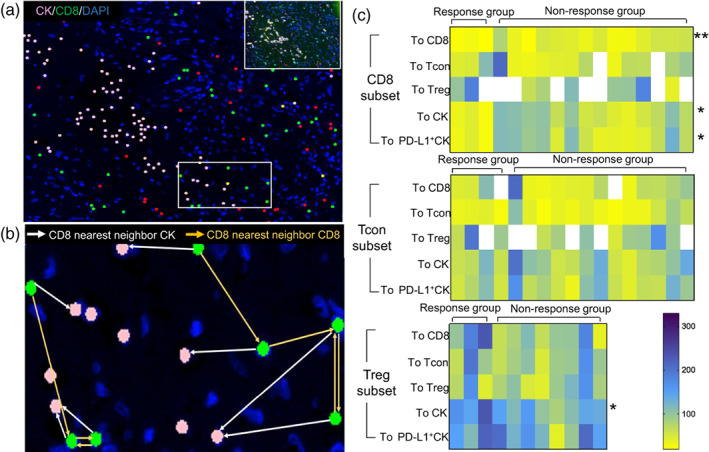
Representative results of spatial analysis and heat map in the response and nonresponse groups. (a) Cell phenotype dot plots of CK (pink)/CD8 (green)/DAPI (blue). (b) Example calculation of the nearest neighbor distance between CD8^+^ T cells and CK cells (white arrows) and between CD8^+^ T cells and CD8^+^ T cells (yellow arrows). (c) Heat map listing the individual distances in the response and nonresponse groups in the CT classified by CD8, CD4^+^Foxp3^−^ conventional T cells (Tcon), and CD4^+^Foxp3^+^ regulatory T cells (Treg). Distance (μm) was plotted from the minimum value (yellow) to the maximum value (dark blue). The *p*‐value was calculated using the Mann–Whitney U test, *p < 0.05, **p < 0.01.

To further examine the role of the TME components, we evaluated the spatial distribution of cells in the TME of pleural mesothelioma. One challenging of using intercellular distances is that the distance is apparently shorter in high cell density samples compared with low cell density samples.^29^ To prevent the effects of outliers and to normalize data, samples with a cell density greater than one standard deviation higher or lower than the mean value in the cohort were excluded (Figure S4). In this analysis, the term “cluster” was introduced to define the nearest neighbor distance within the same cell phenotype; for example, the CD8 cluster referred to the shortest distance between CD8^+^ T cells and their nearest neighbor (Figure 4a, b). CD8^+^ T cells in the CT were more clustered (24.8 μm vs. 61.2 μm, *p* < 0.05) and located closer to CK^+^ tumor cells (39.4 μm vs. 104.6 μm, *p* < 0.05) in the response group than in the nonresponse group (Figure 4c). By contrast, Tregs were located farther away from tumor cells in the CT (221.0 μm vs. 164.8 μm, *p* < 0.05) in the response group than in the nonresponse group (Figure 4c). Closeness between Tregs and CD8^+^ T cells, which is a negative predictor of ICI efficacy,^15^ was not observed in this study. There were no differences regarding of the spatial analysis in the IM (data not shown).

### CD8 cluster demonstrated a strong predictive value in multivariate analysis

To determine the immune‐related parameters such as the density of CD8^+^ T cells and the distance between CD8^+^ T cells and tumor cells, survival analysis was performed after stratifying for these variables to examine the effect of nivolumab on PFS (Figure S5). The closer CD8 cluster was significantly correlated with better PFS (*p* = 0.038). There were no differences in the PFS according to the density of CD8^+^ T cells or CD8^+^ T cells/Tregs to tumor cell distance. The multivariate Cox regression analysis showed that the CD8 cluster was identified as an independent predictor with nivolumab PFS compared with age (Table 3).

**TABLE 3:** Univariate and multivariate Cox regression analysis of PFS in response to nivolumab.

	Univariate	Multivariate			
Variables	*p*‐value	*p*‐value	Hazard ratio	Lower (95% CI)	Upper (95% CI)
Age (≥70 vs. <70)	**0.054**	0.837	1.151	0.303	4.369
Histology (biphasic vs. epithelial)	0.464				
CD8 density (above vs. below average)	0.290				
CD8 to CK distance (above vs. below average)	0.363				
Treg to CK distance (above vs. below average)	0.652				
CD8 cluster (above vs. below average)	**0.008**	**0.021**	7.884	1.366	45.490

*Note*: Bold indicates statistically significant results.

Abbreviations: CI, confidence interval; PFS, progression‐free survival.

## DISCUSSION

In this study, we investigated the distribution of TILs in the TME as well as PD‐L1 expression, using a six‐color mIF panel. The results of quantitative and spatial analyses identified an association between the TME and the response to nivolumab in pleural mesothelioma. The quantitative analysis showed that CD8 was the only T cell subtype positively correlated with the response to nivolumab. The spatial analysis showed that the distance between CD8^+^ T cells and tumor cells and the distance between Tregs and tumor cells played inverse roles in predicting the efficacy of nivolumab.

ICIs have recently revolutionized cancer treatment, yielding remarkable results in many solid tumors.^26^ PD‐L1 is a prognostic predictor of the response to ICIs in NSCLC^30^, ^31^; however, its role in pleural mesothelioma has not been analyzed to date. Few studies have analyzed the association between PD‐L1 expression and the therapeutic efficacy of ICIs in pleural mesothelioma.^32^, ^33^ In this study, the efficacy of nivolumab was not correlated with the expression of PD‐L1, suggesting that PD‐L1 expression alone is not sufficient to predict the therapeutic effects in pleural mesothelioma. Furthermore, PD‐L1 immunohistochemistry needs to be optimized because different detection platforms and antibody clones provide variable results regarding PD‐L1 expression.^34^

TILs in the TME are related to the efficacy of ICIs. CD8 can predict the survival as well as the efficacy of ICIs in several cancers,^35^, ^36^, ^37^, ^38^ and this was confirmed for pleural mesothelioma in the present study. These studies suggest that CD8^+^ T cells as cytotoxic T cells could directly target malignant cells, resulting in activated immunity. In addition, certain CD8^+^ T cell subpopulations (such as TCF7^+^CD8^+^ T cells and PD‐1^+^CD8^+^ T cells) show a favorable correlation with survival and response to anti‐PD‐1 therapy.^39^, ^40^, ^41^, ^42^ Therefore, comprehensive categorization of CD8^+^ TILs may be helpful for patient selection and stratification when considering the indication of ICIs. However, despite their contribution to antitumor immunity in the pleural mesothelioma,^43^ conventional T cells in the TME were not necessarily associated with the response to nivolumab in pleural mesothelioma in this study. Likewise, Tregs, which are among the most studied immunosuppressive cells, were not associated with ICI efficacy in this study. Patients in the nonresponse group showed lower CD8 and higher Treg proportions than the response group, although the difference was not significant. Although these ratios can be used to predict survival,^36^, ^44^, ^45^ this relationship could not be verified in the present study (Figure S6a).

Evaluation of cell phenotypes using mIF is an effective tool for elucidating the complexity of the TME,^44^, ^46^ and spatial analysis provides information on the distance between different immune cells within the TME.^47^, ^48^ Consistent with previous research,^23^ a shorter distance between CD8^+^ T cells and tumor cells was positively associated with the efficacy of ICIs in this study, indicating that proximal CD8^+^ T cells recognize tumor cells and promote antitumor activity. By contrast, Tregs were located further from tumor cells in the response group, suggesting that these cells act in a suppressive manner in tumor immunity. A previous study reported that increased tumor cell‐Treg interaction is associated with poor survival,^15^ this is the first study to relate this finding to the response to ICIs. Although the spatial interaction between Tregs and CD8^+^ T cells predicts patient survival,^32^ this was not confirmed in the present study, suggesting that Tregs suppress CD8^+^ T cells through the production of other immunosuppressive factors rather than through direct cell‐to‐cell contact in pleural mesothelioma.

We investigated whether the immunological features of the TME could predict the occurrence of irAEs. We showed that the percentage of CD8^+^ T cells and Tcons among total T cells was associated with irAEs (Figure S6b). Indeed, a previous study reported that an abundance of activated CD4 memory T cells is associated with severe irAEs in melanoma.^49^ A detailed subtype classification of CD4^+^ T cells could be useful to predict the occurrence of irAEs.

The present study had several limitations. Because this is a rare tumor type, the sample size of metastatic pleural mesothelioma patients treated with nivolumab was small. Since there were only four patients in the response group, a larger validation cohort and further careful examination of statistical significance are required. This study only included surgical specimens and there was a lack of biopsy specimens. Moreover, the absence of patients who received chemotherapy may also have been a confounding factor. In addition, mIF staining was limited to a subset of immune cell types. For example, CD20^+^ B cells, natural killer cells, and macrophages in the TME, which are associated with patient survival, were not examined in this study.^43^, ^50^, ^51^, ^52^ Further studies are needed to address these concerns. Finally, this was a retrospective study, and prospective research is needed to validate and improve upon these findings.

In conclusion, we showed that high density and proximity of CD8^+^ T cells, as well as a greater Treg to tumor distance were associated with a better response to nivolumab. Analysis of the spatial distribution of cells using mIF may help predict the efficacy of ICIs. The present findings indicate that the distinct landscape of the TME is associated with a stratified response to ICIs, and analysis of the TME could provide prognostic information on the effect of immunotherapy in pleural mesothelioma.

## AUTHOR CONTRIBUTIONS

Yuting Yin: Conceptualization, methodology, formal analysis, investigation, data curation, writing‐original draft, writing–review and editing, visualization. Rie Sakakibara: Conceptualization, methodology, formal analysis, investigation, resources, data curation, writing‐original draft, writing–review and editing, visualization, project administration. Takayuki Honda: Conceptualization, formal analysis, investigation, resources, data curation, writing‐original draft, writing–review and editing, visualization, project administration. Susumu Kirimura: Methodology, resources, writing–review and editing. Pissacha Daroonpan: Methodology, writing–review and editing. Masashi Kobayashi: Resources, writing–review and editing. Kohei Ando: Resources, investigation, writing–review and editing. Hideki Ujiie: Resources, investigation, writing–review and editing. Tatsuya Kato: Resources, writing–review and editing. Kichizo Kaga: Resources, writing–review and editing. Takahiro Mitsumura: Resources, investigation, writing–review and editing. Ryoji Nakano: Resources, writing–review and editing. Hiroyuki Sakashita: Resources, investigation, writing–review and editing, project administration. Shinichi Matsuge: Resources, investigation, writing–review and editing. Hironori Ishibashi: Resources, investigation, writing–review and editing. Takumi Akashi: Resources, writing–review and editing. Yasuhiro Hida: Resources, investigation, writing–review and editing. Takao Morohoshi: Resources, investigation, writing–review & editing. Miyuki Azuma: Methodology, writing–review and editing, visualization, project administration. Kenichi Okubo: Resources, investigation, writing–review and editing, project administration. Yasunari Miyazaki: Methodology, investigation, writing–review and editing, visualization, supervision, project administration.

## CONFLICT OF INTEREST STATEMENT

R. Sakakibara reports a personal fee from Ono Pharmaceutical Co. Ltd. H. Sakashita reports personal fee as well as institutional fee from *Ono Pharmaceutical Co. Ltd*. Azuma M reports grant from JSPS KAKENHI (JP21H03138). Miyazaki Y reports YM endowed chair (personal fee) from *Ono Pharmaceutical Co. Ltd*. For the remaining authors none were declared.

## Supporting information


**Figure S1:** Definition of invasive margin (IM) and central tumor (CT). (a) CK staining and DAPI staining at 40× magnification. (b) Representative images of intra‐ and extratumoral areas. The intratumor areas were defined as the CT, and the extratumoral areas were defined as the IM. Images are shown at 200× magnification.
**Figure S2:** Representative microphotograph of multiplex immunofluorescence (mIF), phenotyping algorithm, and cell phenotype colocalization in pleural mesothelioma at 200× magnification. (a) Representative mIF six‐color composite image of an pleural mesothelioma surgical specimen. Fluorescence: CK (pink)/PD‐L1 (cyan)/CD8 (green)/CD4 (red)/Foxp3 (yellow)/DAPI (blue). (b) Cell phenotype evaluated by training procedures in inForm analysis software. (c) Representative marker expression of malignant cells (CK) and colocalization with PD‐L1. (d) Representative marker expression of CD4^+^Foxp3^−^ indicates conventional T cells (Tcons) and colocalization of CD4^+^ and Foxp3^+^ as regulatory T cells (Tregs).
**Figure S3:** Comparison of 22C3 and E1J2J clones of PD‐L1 antibodies. (a) Graph of the correlation between two PD‐L1 antibodies (clone E1J2J and clone 22C3). The *p* value was calculated by Spearman's correlation. (b) Representative staining of clone E1J2J; PD‐L1 TPS was 30%. (c) Representative staining of clone 22C3; PD‐L1 TPS was 30%. (b,c) Consecutive FFPE slides from the same patient were selected. Images are shown at 200× magnification.
**Figure S4:** Normalization of CD8^+^ T cell density. In the spatial analysis, outliers showing one standardized deviation higher or lower than the mean were excluded from the study. The red line indicates the mean CD8 density.
**Figure S5:** Survival analysis of potential predictors identified cluster as significant. Kaplan–Meier curves of CD8 density (a), CD8 to CK distance (b), Treg to CK distance (c), and CD8 to CD8 distance (CD8 cluster) (d) between the response and nonresponse groups. A mean cutoff was used to separate the high and low groups. The *p*‐value, HR, and 95% CI were calculated by a log‐rank test. Abbreviations: CD8, CD8^+^ T cells; Tregs, regulatory T cells; hi, high; lo, low.
**Figure S6:** Ratio analysis and the correlation between immune‐related adverse event (irAE) occurrence and three T cell subpopulations. (a) Ratios of T cell subsets in the central tumor and invasive margin were analyzed in the response and nonresponse groups. (b) Proportions of 3 T‐cell subsets in patients with ≥grade 3 toxicity and no toxicity patients. The *p*‐value was calculated using the Mann–Whitney U test, *p < 0.05. Abbreviations: ns, no significance; Tcon, conventional T cells; Treg, regulatory T cells.
