# Supplementary material for: High density and proximity of CD8 + T cells to tumor cells are correlated with better response to nivolumab treatment in metastatic pleural mesothelioma
Source: Thorac Cancer. 2023 May 30;14(20):1991–2000. doi: 10.1111/1759-7714.14981 (PMC10344741; doi:10.1111/1759-7714.14981)
Supplement: Supplementary file 1 — Figure S1: Definition of invasive margin (IM) and central tumor (CT). (a) CK staining and DAPI staining at 40× magnification. (b) Representative images of intra‐ and extratumoral areas. The intratumor areas were defined as the CT, and the extratumoral areas were defined as the IM. Images are shown at 200× magnification. Figure S2: Representative microphotograph of multiplex immunofluorescence (mIF), phenotyping algorithm, and cell phenotype colocalization in pleural mesothelioma at 200× magnification. (a) Representative mIF six‐color composite image of an pleural mesothelioma surgical specimen. Fluorescence: CK (pink)/PD‐L1 (cyan)/CD8 (green)/CD4 (red)/Foxp3 (yellow)/DAPI (blue). (b) Cell phenotype evaluated by training procedures in inForm analysis software. (c) Representative marker expression of malignant cells (CK) and colocalization with PD‐L1. (d) Representative marker expression of CD4+Foxp3− indicates conventional T cells (Tcons) and colocalization of CD4+ and Foxp3+ as regulatory T cells (Tregs). Figure S3: Comparison of 22C3 and E1J2J clones of PD‐L1 antibodies. (a) Graph of the correlation between two PD‐L1 antibodies (clone E1J2J and clone 22C3). The p value was calculated by Spearman's correlation. (b) Representative staining of clone E1J2J; PD‐L1 TPS was 30%. (c) Representative staining of clone 22C3; PD‐L1 TPS was 30%. (b,c) Consecutive FFPE slides from the same patient were selected. Images are shown at 200× magnification. Figure S4: Normalization of CD8+ T cell density. In the spatial analysis, outliers showing one standardized deviation higher or lower than the mean were excluded from the study. The red line indicates the mean CD8 density. Figure S5: Survival analysis of potential predictors identified cluster as significant. Kaplan–Meier curves of CD8 density (a), CD8 to CK distance (b), Treg to CK distance (c), and CD8 to CD8 distance (CD8 cluster) (d) between the response and nonresponse groups. A mean cutoff was used to separate the high and lo [file TCA-14-1991-s001.pptx]

## Slide 1
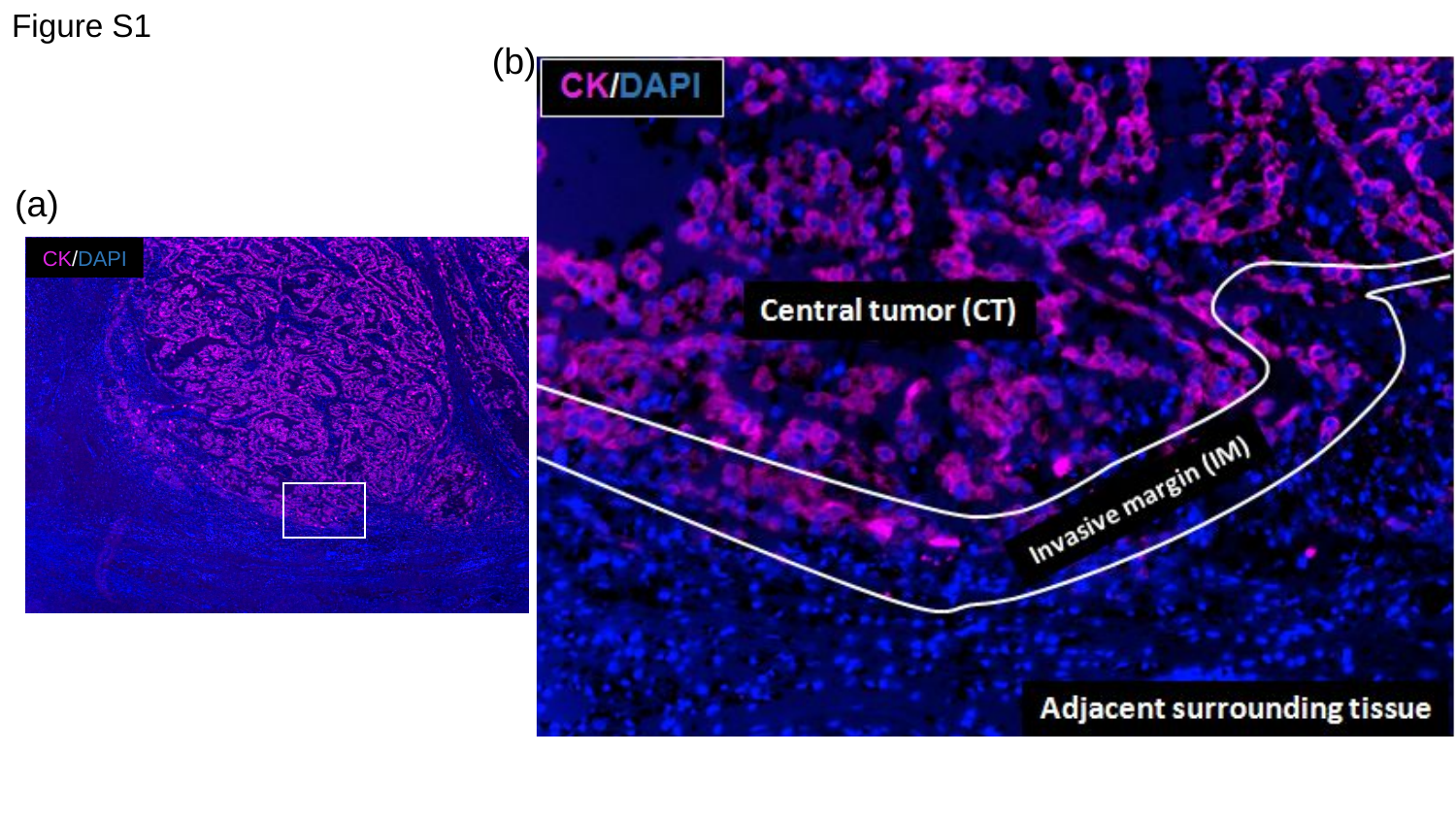

Figure S1
(b)
(a)
CK/DAPI

## Slide 2
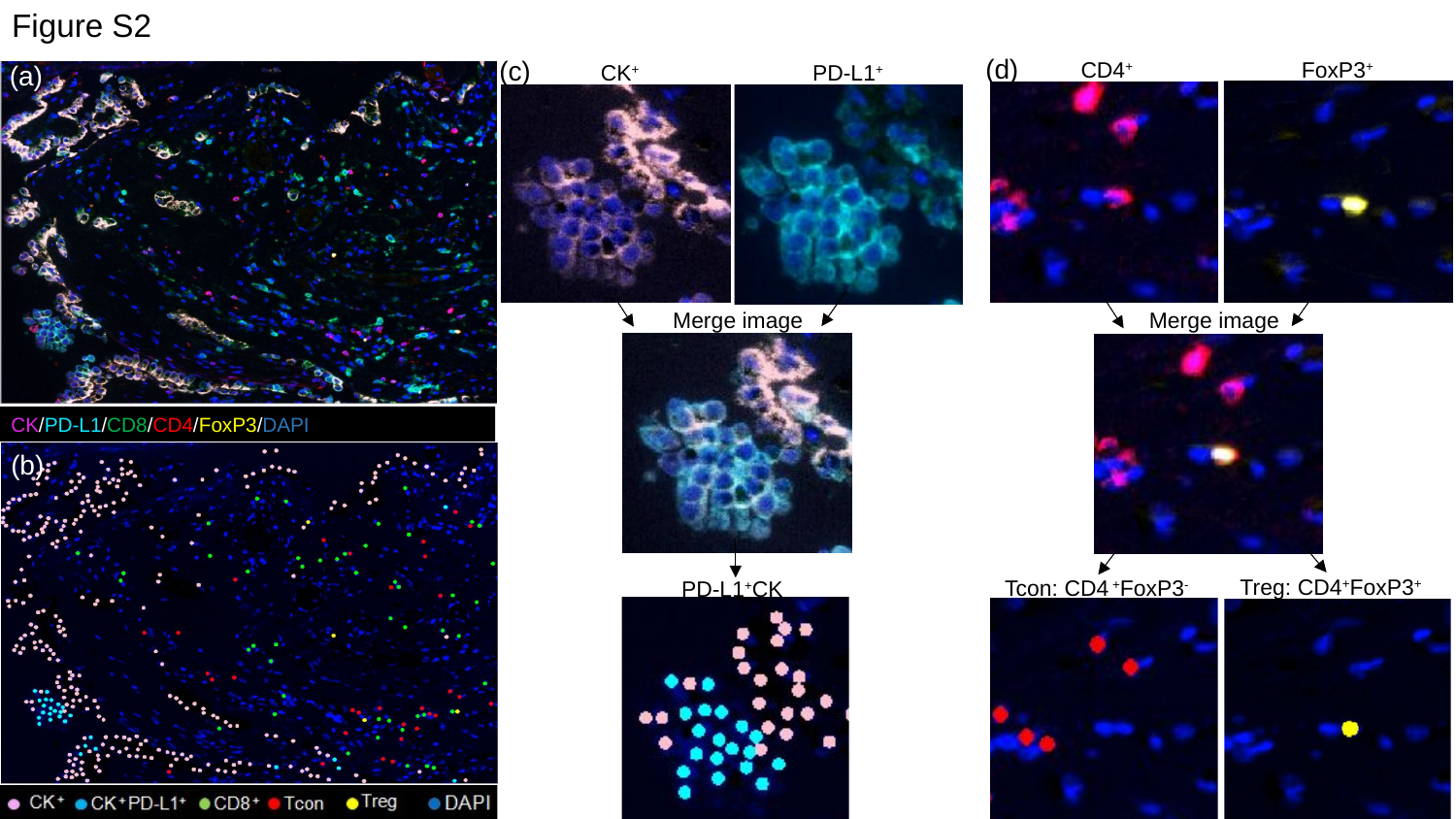

Figure S2
(d)
(c)
CD4+
FoxP3+
(a)
CK+
PD-L1+
Merge image
Merge image
CK/PD-L1/CD8/CD4/FoxP3/DAPI
(b)
Treg: CD4+FoxP3+
Tcon: CD4 +FoxP3-
 PD-L1+CK

## Slide 3
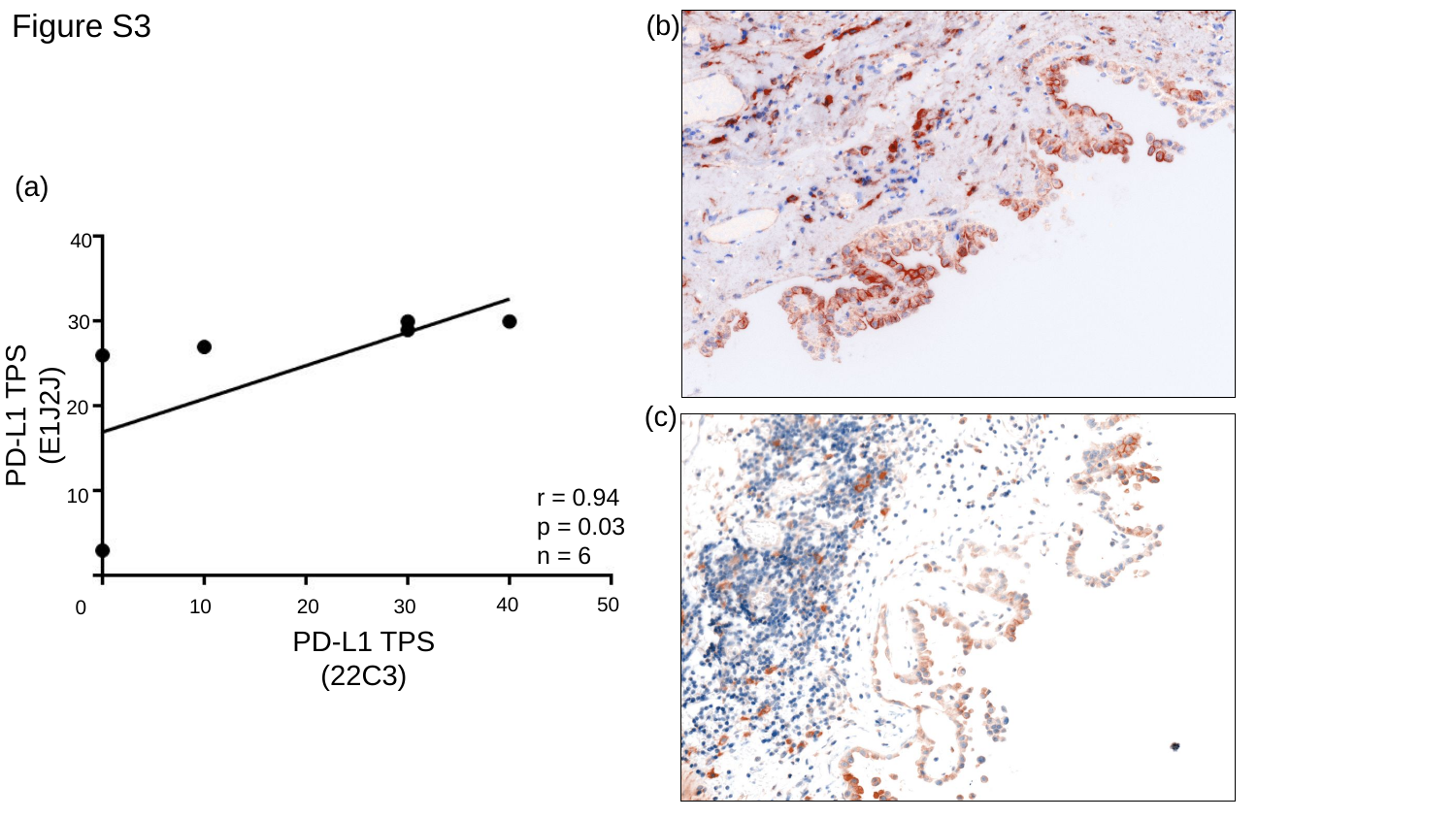

Figure S3
(b)
(a)
40
30
PD-L1 TPS
(E1J2J)
20
(c)
r = 0.94
p = 0.03
n = 6
10
40
50
10
20
30
0
PD-L1 TPS
(22C3)

## Slide 4
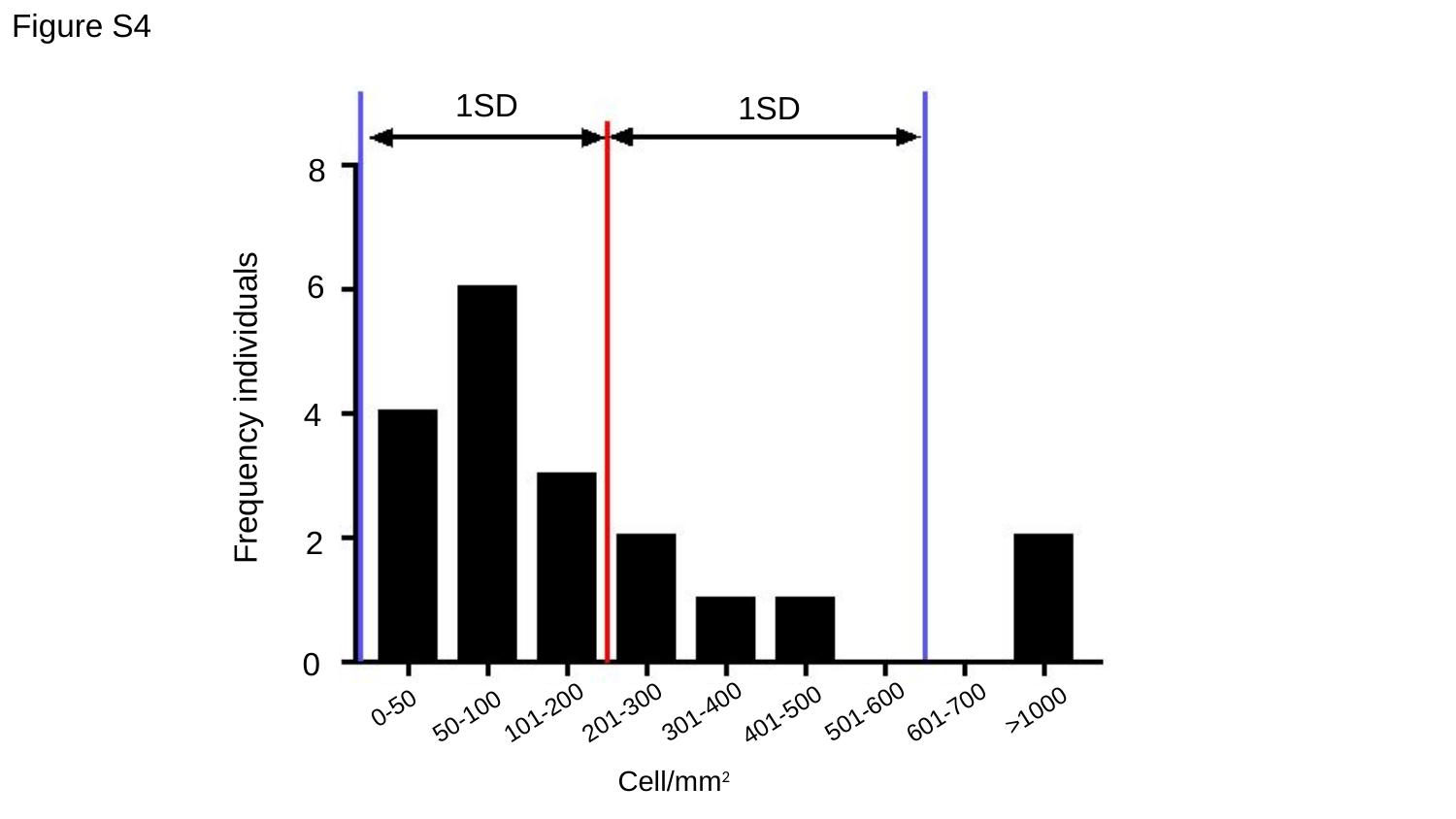

Figure S4
1SD
1SD
8
6
Frequency individuals
4
2
0
0-50
>1000
501-600
301-400
201-300
101-200
601-700
401-500
50-100
Cell/mm2

## Slide 5
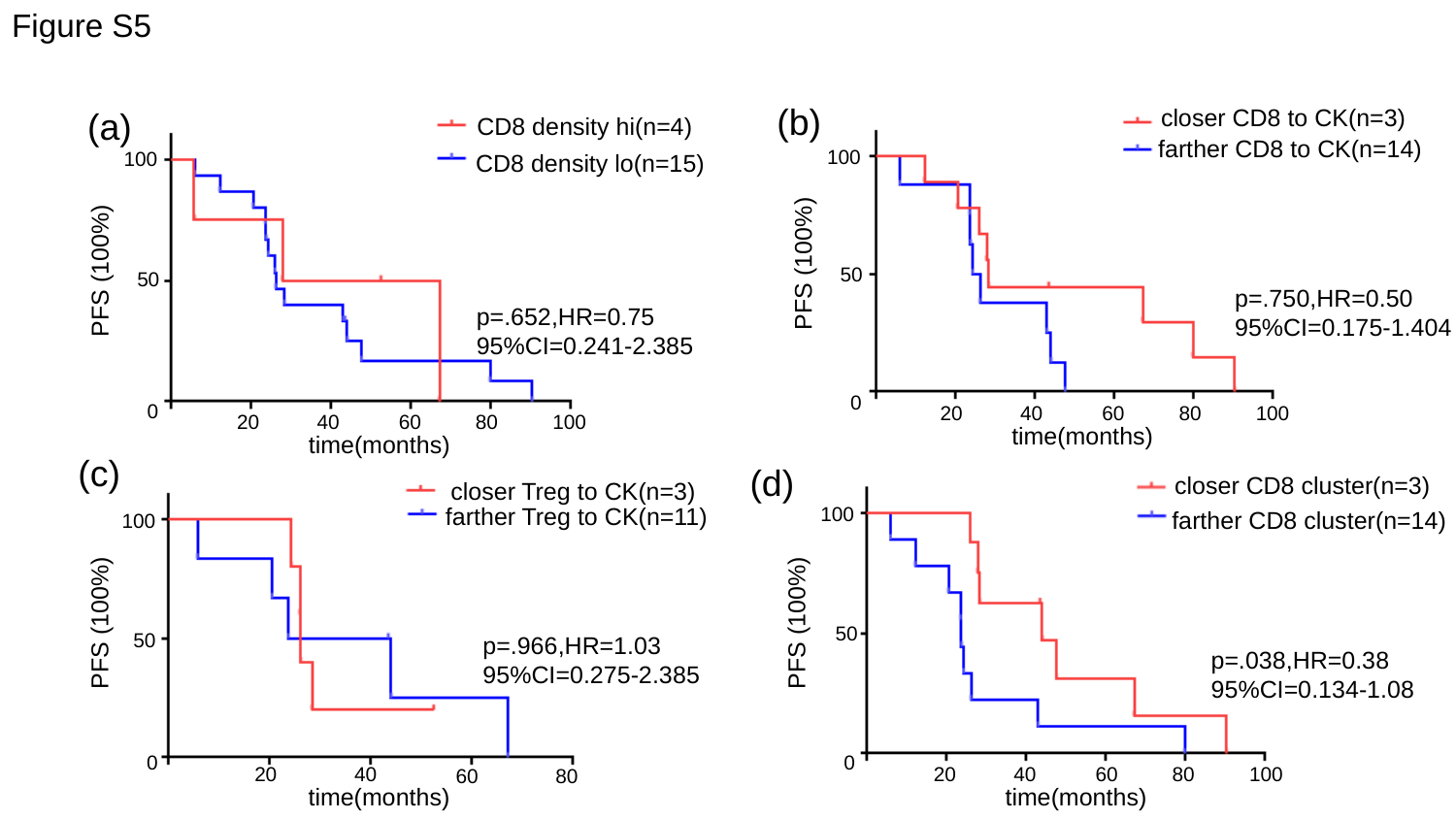

Figure S5
(b)
closer CD8 to CK(n=3)
(a)
CD8 density hi(n=4)
farther CD8 to CK(n=14)
100
100
CD8 density lo(n=15)
PFS (100%)
PFS (100%)
50
50
p=.750,HR=0.50
95%CI=0.175-1.404
p=.652,HR=0.75
95%CI=0.241-2.385
0
0
20
40
60
80
100
20
40
60
80
100
time(months)
time(months)
(c)
(d)
closer CD8 cluster(n=3)
closer Treg to CK(n=3)
100
farther Treg to CK(n=11)
farther CD8 cluster(n=14)
100
PFS (100%)
PFS (100%)
50
50
p=.966,HR=1.03
95%CI=0.275-2.385
p=.038,HR=0.38
95%CI=0.134-1.08
0
0
20
40
20
40
60
80
100
60
80
time(months)
time(months)

## Slide 6
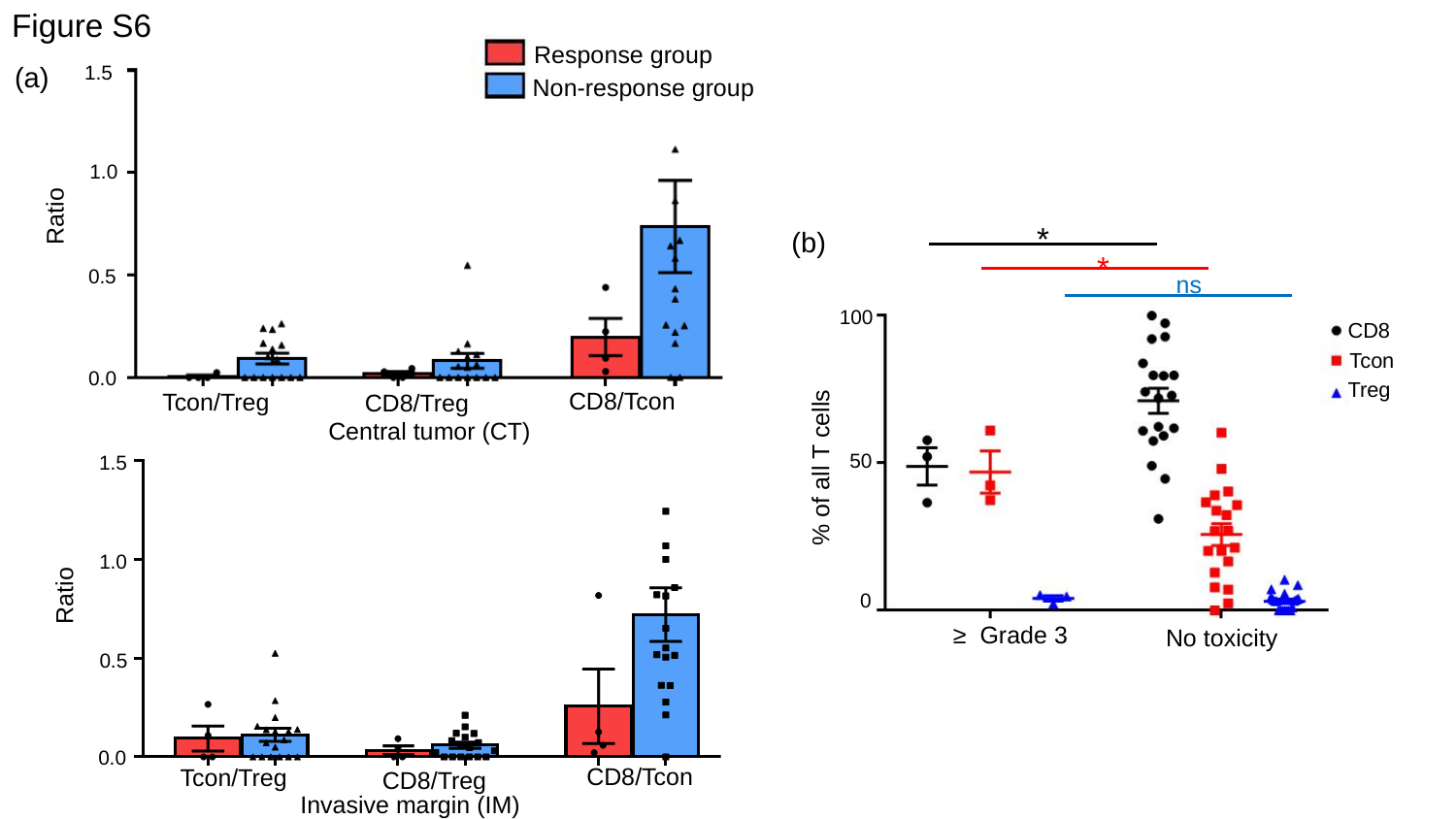

Figure S6
Response group
(a)
1.5
Non-response group
1.0
Ratio
*
(b)
*
0.5
ns
100
CD8
Tcon
0.0
Treg
CD8/Tcon
Tcon/Treg
CD8/Treg
Central tumor (CT)
50
1.5
% of all T cells
1.0
Ratio
0
≥ Grade 3
No toxicity
0.5
0.0
CD8/Tcon
Tcon/Treg
CD8/Treg
Invasive margin (IM)
